# Characterization of an Ebosin derivative produced by heterologous gene replacement in *Streptomyces* sp. 139

**DOI:** 10.1186/s12934-014-0103-6

**Published:** 2014-07-22

**Authors:** Yang Zhang, Junjie Shan, Yonggang Bao, Liping Bai, Rong Jiang, Lianhong Guo, Chen Yao, Ren Zhang, Yuan Li

**Affiliations:** 1 Key laboratory of Biotechnology of Antibiotics, Ministry of Health, Institute of Medicinal Biotechnology, Chinese Academy of Medical Sciences & Peking Union Medical College, Tian Tan, Beijing, 100050, China; 2 Institute of Pharmacology and Toxicology, Taiping Road, Beijing, 100850, China; 3 School of Biological Sciences, University of Wollongong, Wollongong 2522, NSW, Australia

**Keywords:** Ebosin derivative, Heterologous gene replacement, Gene gtf, Gene ste7, Streptomyces, Streptococcus thermophilus

## Abstract

**Background:**

Ebosin is a novel exopolysaccharide (EPS) produced by *Streptomyces* sp. 139 and evidenced to possess an anti-rheumatic arthritis activity *in vivo.* The Ebosin biosynthesis gene cluster (*ste*) consists of 27 ORFs and *ste7* has previously been demonstrated to code for a fucosyltransferase, which plays an essential role in the formation of repeating sugar units during Ebosin production. Aiming to generate derivatives of Ebosin for better activity, we replaced *ste7* with a gene encoding for a glucosyltransferase (*gtf*) from *Streptococcus thermophilus.*

**Results:**

This alteration resulted in a novel Ebosin derivative (EPS-7 g) with its monosaccharide composition dramatically changed, especially in the proportion of glucose which increased from 1.1% (Ebosin) to 84.01% (EPS-7 g). In an ELISA analysis, EPS-7 g exhibited a higher binding activity for IL-1R, as a competitor of interleukin-1, than that of Ebosin. It also exhibited a higher inhibitory effect on the activity of IL-1β-converting enzyme and production of IL-1β in fibroblast-like synoviocytes (FLS). In addition, experiments with acute inflamed mice induced by croton oil showed a significantly higher anti-inflammatory activity of EPS-7 g compared with Ebosin.

**Conclusions:**

The new Ebosin derivative EPS-7 g is more bioactive than Ebosin evaluated by a series of experiments*.* This is the first report demonstrating a modification of EPS structure via heterologous gene replacement in *Streptomyces*.

## Background

One type of microbial polysaccharides are secreted out of the cells [1] and therefore called exopolysaccharides (EPSs), which are long-chain polysaccharides consisting of branched, repeating units of sugars or sugar derivatives [2]. In bacteria, EPS biosynthesis starts with the intracellular formation of EPS precursors and the sugar nucleotides, followed by the formation of a repeating unit on a lipid carrier which is located in the cytoplasmic membrane. The later steps involve transport of the repeating units across the membrane to the outer layer and polymerization of tens and even hundreds of such units to form the final EPSs [3]. During the process, glycosyltransferases play important roles to sequentially transfer sugars from intracellular nucleotide sugars to a lipid carrier acceptor [4].

In order to improve applications of EPSs, genetic engineering can be used in the production of desired polysaccharides targeting predefined macroscopic properties.

Based on the available genetic information, genetic modification of *eps* genes should lead to EPSs with a different repeating unit or with a different chain length [5]. Manipulation of genes which function in export, polymerization, and determination of chain length during EPS biosynthesis were also proved effective for altering EPS structure [6]. Heterologous production of an EPS has been successful by transfer of the complete *eps* gene cluster alone of a LAB strain into a non EPS-producing heterologous host, provided that the heterologous host possessed all necessary genetic information for precursor synthesis [7]. Ingeborg et al [8] described increased exopolysaccharide production in *Lactoccus lactis* due to manipulated overexpression of the NIZO B40 *eps* gene cluster, the first report demonstrating that homologous overexpression of a complete *eps* gene cluster in *Lactoccus lactis* leads to increased EPS production.

*Streptomyces* are a group of gram-positive bacteria that have been intensively studied for their secondary metabolites, particularly antibiotics. However, little is known of the production of EPSs in *Streptomyces*. Recently a novel EPS namely Ebosin was isolated from the supernatants of fermentation cultures of *Streptomyces* sp. 139 [9], which has remarkable anti-rheumatic arthritis activity in *vivo*[10]. Its biosynthesis gene cluster (*ste*) consisting of 27 ORFs was also identified [11]. Efforts have been made in elucidating the function of individual *ste* genes [12]–[15]. Insights into the biosynthesis pathways of polysaccharides are crucial for the exploitation of microorganisms to produce polysaccharides of industrial or medicinal importance. With a number of the *ste* genes identified, it is now possible to carry out specific manipulations for creating Ebosin derivatives which can then be screened for better bioactivities. This paper reports our endeavor to generate such derivatives by tackling the *ste7* gene encoding a fucosyltransferase, which catalyzes the transfer of fucose specifically from GDP*-*fucose to a fucose acceptor during Ebosin biosynthesis [16]. In this study, we replaced *ste7* with the glucosyltransferase gene (*gtf*) from *Streptococcus thermophilus*, which is a gram-positive bacterium and most valuably used in food industry. Stingele et al [17] identified the *eps* genetic locus of *S. thermophilus* Sfi6, revealing a 15.25-kb region containing 16 open reading frames (ORFs), within a 14.52-kb region encodes 13 genes (*epsA to epsM*) capable of directing EPS synthesis. The resultant EPS-7 g produced by the strain *Streptomyces* sp. 139 (*gtf*) has a very high content of glucose and exhibited remarkably higher bioactivities than that of Ebosin in both *in vitro* and *in vivo* assays.

## Results

### Construction of the heterologous gene replacement strain *Streptomyces* sp*.* 139 *(gtf)*

**Figure 1:**
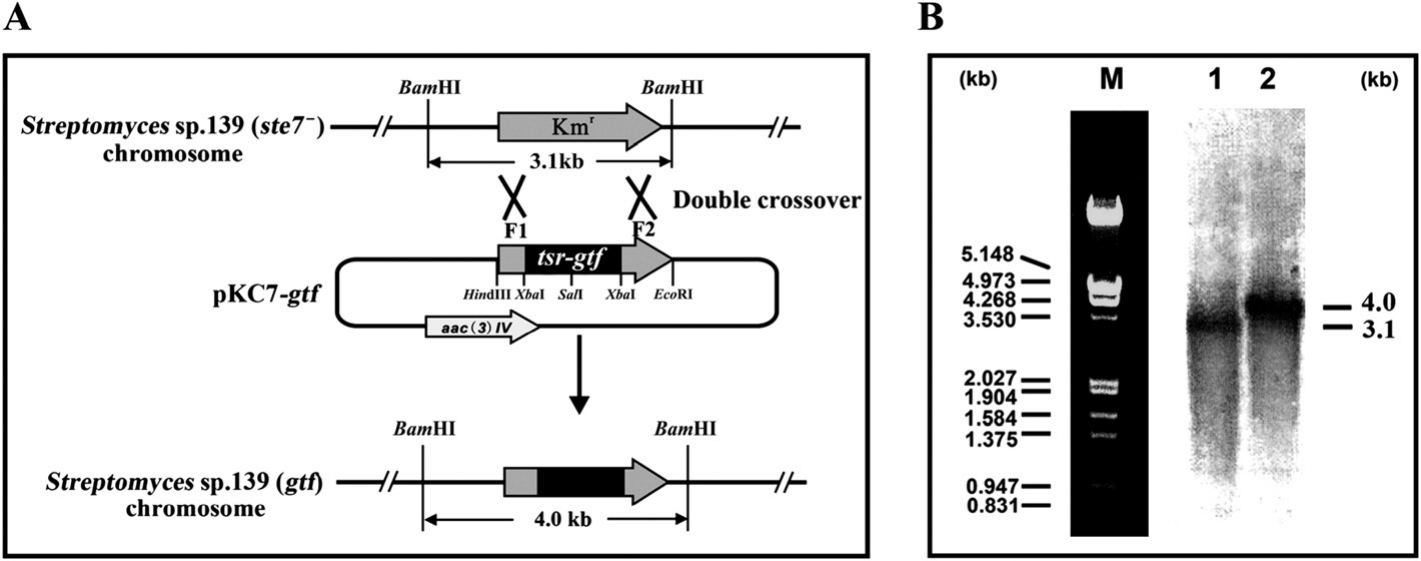
**The diagram of gene replacement and Southern blot analysis. (A)** Diagram of gene replacement of *ste7* with *gtf* originated from *Streptococcus thermophilus* through a double crossover via homologous recombination. Gray box indicates the location of Km^r^ gene in *Streptomyces* sp. 139 (*ste7*^***-***^). Restriction maps of *Streptomyces* sp. 139 (*ste7*^***-***^) and the gene replacement strain *Streptomyces* sp. 139 (*gtf*) show the predicted fragment sizes upon *Bam*HI digestion. **(B)** Southern blot autoradiograph of *Streptomyces* sp. 139 (*ste7*^***-***^) and *Streptomyces* sp. 139 (*gtf*). 1. Chromosome DNA of *Streptomyces* sp. 139 (*ste7*^***-***^) digested with *Bam*HI; 2. chromosome DNA of *Streptomyces* sp. 139 (*gtf*) digested with *Bam*HI*.*

The replacement of *ste7* gene by the *gtf* gene of *S. thermophilus* (Figure 1A) was evidenced by Southern hybridization using a 1.03-kb DNA (F1) as probe upstream of *ste7* (Figure 1B). The hybridization signals appeared with the expected sizes of 4.0 kb for *Streptomyces* sp. 139 (*gtf*) and 3.1 kb for *Streptomyces* sp. 139 (*ste7*^-^). This result indicated that the colonies with thio^r^ Am^s^ phenotypes had integrated the *gtf*-*thio*^*r*^ cassette into the kanamycin resistance gene, therefore, confirmed the establishment of heterologous gene replacement strain *Streptomyces* sp. 139 (*gtf*).

### Sugar composition of EPSs

**Figure 2:**
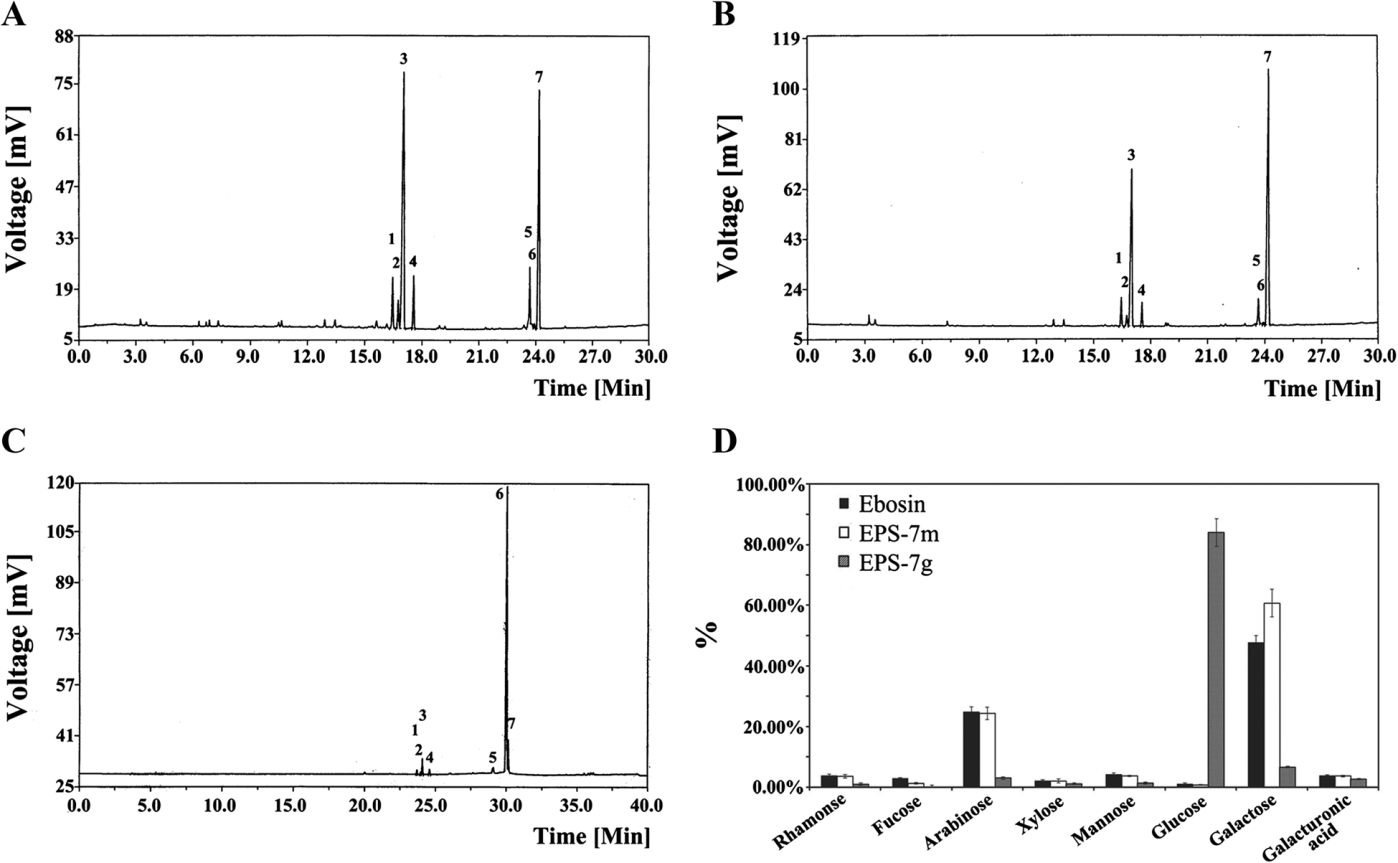
**GC chromatograms of sugar analysis of Ebosin (A), EPS-7 m (B) and EPS-7 g (C).** 1. rhamnose, 2. fucose, 3. arabinose, 4. xylose, 5. mannose, 6. glucose, 7. galactose. **(D)**. Monosaccharide compositions of Ebosin, EPS-7 m and EPS-7 g. Galacturonic acid was analyzed using a method based on uronic acid carbazole reaction (Bitter *et al.,* 1962).

GC analysis of Ebosin, EPS-7 m produced by the knock mutant *Streptomyces* sp. 139 (*ste7*^-^) and EPS-7 g by *Streptomyces* sp. 139 (*gtf*) (Figure 2A, B, C) showed that these EPSs consisted of the same monosaccharides: glucose, mannose, arabinose, galactose, fucose, xylose and rhamnose, of which the most striking was an increase of glucose proportion from 1.1% (Ebosin) to 84.01% (EPS-7 g). While, some proportional changes of other sugars also occurred in comparison with Ebosin (Figure 2D). Using a method based on uronic acid carbazole reaction, galacturonic acid was also found in EPS-7 m and EPS-7 g.

### The competitive binding activity of Ebosin derivates with IL-1 for IL-1R

Using an ELISA assay, the competitive binding activities of Ebosin, EPS-7 m and EPS-7 g with IL-1 for IL-1R were determined.

**Figure 3:**
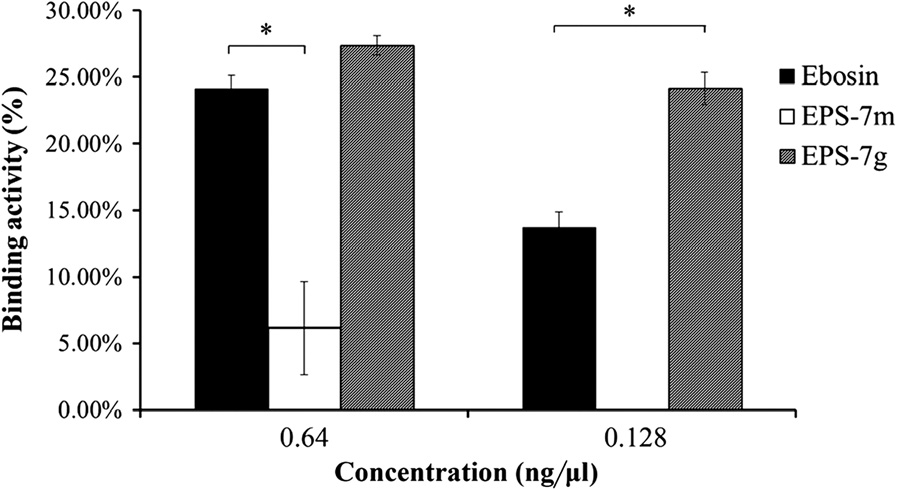
Binding activities of Ebosin, EPS-7 m and EPS-7 g to IL-1R determined by an ELISA assay.

For EPS-7 m they were 6.1% and nil (*P* < 0.05) at dosages of 0.64 ng/μL and 0.128 ng/μL respectively, which were remarkably lower than those of Ebosin (24.2%, 13.7%) at the same dosages. Higher binding activities of EPS-7 g were detected to be 27.1% and 24.3% (*P* < 0.05) respectively compared with that of Ebosin at same concentrations (Figure 3).

### Effect of Ebosin derivates on the enzymatic activity for IL-1β-converting enzyme (ICE)

**Figure 4:**
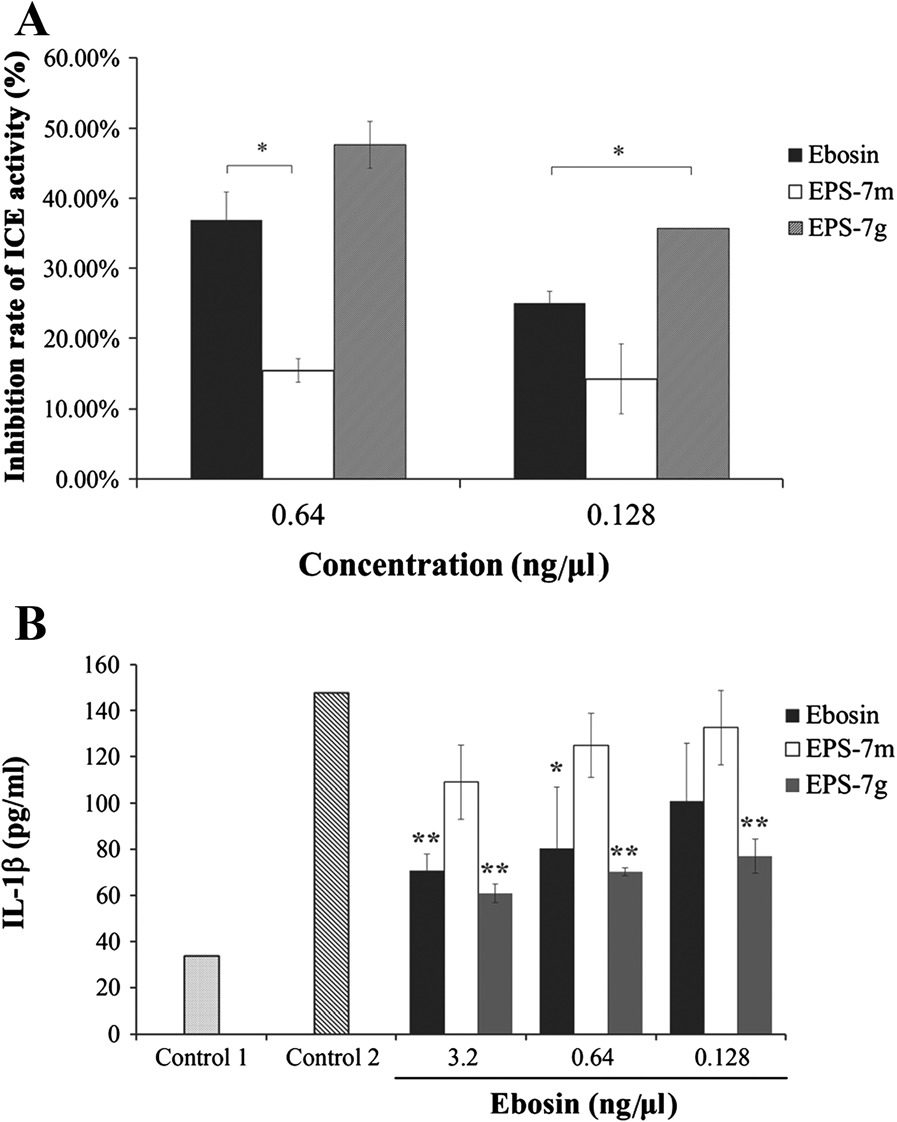
**Suppression of ICE enzymatic activity (A) and inhibited production of IL-1β in FLS (B) by Ebosin, EPS-7 m and EPS-7 g.** *, *P < 0.05;* **, *P < 0.01*; ***, *P < 0.001* versus FLS without stimulating with LPS (control 1).

At concentrations of 0.64 ng/μL and 0.128 ng/μL, Ebosin suppressed the enzyme with inhibition ratios 35.5% and 25.0% respectively, compared with 15.5% (*P* < 0.05), 14.3% for EPS-7 m and 47.6%, 35.7% (*P* < 0.05) for EPS-7 g respectively (Figure 4A). The results demonstrated a higher activity of EPS-7 g to inhibit the IL-1β-converting enzyme.

### Inhibiting effect of Ebosin derivates on production of IL-1β in FLS cells

To assess the effects of Ebosin, EPS-7 m and EPS-7 g on production of IL-1β in FLS (fibroblast-like synoviocytes), cell cultures were carried out and stimulated with LPS at 37°C for 72 h before analyzed by ELISA. The results indicated (Figure 4B) that Ebosin, EPS-7 m and EPS-7 g at dosage of 3.2 ng/μL reduced the IL-1β production 52.03% (*P* < 0.01), 26.17%, 58.70% (*P* < 0.01) respectively and 45.57% (*P* < 0.5), 15.39%, 52.34% (*P* < 0.01) at dosage of 0.64 ng/μL separately. With dosage of 0.128 ng/μL, the IL-1β production were suppressed 31.61%, 10.16% and 47.82% (*P* < 0.01) individually.

### Effect of Ebosin and EPS-7 g on the inflammatory activity in the acute inflamed mice induced by croton oil

**Figure 5:**
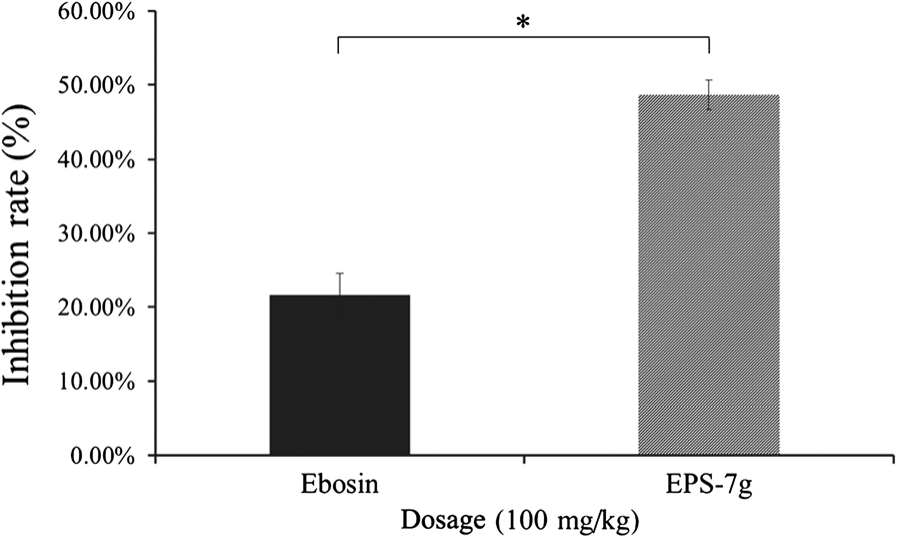
Effect of EPSs on inflammatory activity in the acute inflamed mice induced by croton oil.

To evaluate the effect of Ebosin and EPS-7 g on the acute inflamed mice induced by croton oil, the mice were randomly divided into three groups described as above. Ebosin and EPS-7 g (100 mg/kg) were orally administered to each mouse of the respective experimental groups for 1 h before induction by croton oil. Compared with the acute inflamed mice groups untreated (control), the inhibition ratios of EPS-7 g and Ebosin for mice ear edema were 48.61% (*P* < 0.05) and 21.60% respectively (Figure 5), indicating that EPS-7 g surpasses Ebosin by over 100% on suppressing the inflammatory activity in the acute inflamed mice.

## Discussion

Exopolysaccharides include a range of diverse polymers that play vital roles in variety of biological processes. In addition, EPSs have also significant industrial applications, including their use as biothickeners in foods [4]. Notably, EPSs produced by lactic acid bacteria contribute significantly to the structure and viscosity of fermented milk products [18]. The health value of these macromolecules has also emerged in recent years; many reports indicate that they can confer health benefits on consumers arising from their immunogenic and cholesterol-lowering properties [19],[20]. EPSs produced by *Trichoderma erinaceum* DG-312 was shown to have a strong anti-inflammatory activity in inflamed mice [21]. *Enterobacter cloacae* was also found to produce EPSs with anti-diabetic activity [22].

Metabolic engineering has enabled generation of “designer” polysaccharides in lactic acid bacteria (LAB), which mostly involved manipulations of glycosyltransferases [2]. Introducing new or existing glycosyltransferases into LAB [4] or gene shuffling with glycosyltransferases are effective means for controlling EPS structure [5]. Masja et al [23] reported that heterologous production of the pneumococcal serotype 14 polysaccharide in *Lactococcus lactis* resulted in the recombinant product secreted into culture medium, which simplified downstream processing. This was achieved by coexpressing the pneumococcal gene cluster *cpsFGHIJKL*_14_ with the lactococcal regulatory and priming glucosyltransferase-encoding genes (*epsABCD*_B40_) specific for B40 polysaccharide. Knoshaug et al [24] reported evidence for a novel gene organization expressing EPS in *Lactococcus lactis* subsp. *cremoris* Ropy352 and showed the specificities of polymerization and export enzymes blocking the function of just one glycosyltransferase abolished the production of ropy EPS. Heterologous expression of glycosyltransferases has been found to result in different sugars added at strategic positions in generating EPSs with new properties [5].

Ebosin is a novel EPS with anti-rheumatic arthritis activity. It has been shown that Ebosin is an inhibitor of IL-1β-converting enzyme (ICE), a key enzyme in synthesis of IL-1β [25]. More recently, evidence has also been obtained demonstrating that the anti-inflammatory effect of Ebosin on rat collagen-induced arthritis is through suppressing production of interleukin-1β, interleukin-6 and tumor necrosis factor α at both transcriptional and posttranslational levels [10]. This EPS is therefore of medicinal value so improvement for better property is warranted. In this study, a glucosyltransferase *gtf* gene from *S. thermophiles* was expressed in replacement of the *ste7* gene which encodes a fucosyltransferase in the Ebosin-producing strain *Streptomyces* sp. 139. This brought about dramatic changes in the property of EPS produced. Because of the complexity of the biosynthesis pathway of Ebosin and involvement of a large number of genes, once *ste7* was replaced with the heterologous gene *gtf*, dramatic effect was seen not only on the increased incorporation of glucose, but also on the overall sugar profile. The reason for these changes, which are apparently not a simple proportional change of composition with increased glucose, may lie in the primary structure. The resultant EPS-7 g turns out to be more bioactive compared with Ebosin evaluated by its ICE inhibitory activity, suppression of interleukin-1β production in fibroblast-like synoviocytes (FLS), competitive binding to IL-1R against IL-1 and suppression of the acute inflammatory activity in the acute inflamed mice induced by croton oil. According to these results, it looks like that increasing proportion of glucose effects on bioactivity of EPS-7 g. A good understanding of the correlation between structure and bioactivity of Ebosin is important because it will provide a foundation for a strategy aimed at producing functionally more valuable polysaccharides. Continuing efforts in the elucidation of Ebosin biosynthesis pathway will enable more rational designs for genetic manipulation to generate highly effective derivatives.

## Conclusions

EPS-7 g, a novel Ebosin derivate, was produced by heterologous gene replacement in *Streptomyces* sp. 139, which was more bioactive compared with Ebosin evaluated by a series of experiments. To our knowledge, this is the first report on manipulation of EPS structure by introducing heterologous glucosyltransferases into *Streptomyces*.

## Methods

### Bacterial strains and culture conditions

*Streptomyces* sp. 139 was isolated from a soil sample in China and kept in the China General Microbiology Culture Collection Center (No. 0405) (Table 1). *Streptomyces* sp. 139 (*ste7*^-^) was generated previously [16] and kept in our laboratory. These strains were cultured at 28°C with shaking (250 rpm) in either TSB medium supplemented with 5 mM MgCI_2_ and 0.5% glycine or fermentation medium (1% glucose, 2% starch, 2% soybean extract, 0.2% tryptone, 0.2% beef extract, 0.4% yeast extract, 0.05% K_2_HPO4, 0.3% CaCO_3_, pH 7.3). *Streptococcus thermophilus* was obtained from China Industrial Microbiology Culture Collection Center (CICC 20370) (Table 1)and cultured in LB medium at 40°C.

**Table 1:** Bacterial strains and plasmids used in this study

**Strain or plasmid**	**Description**	**Reference**
**Strains**		
*E.coli* DH5α	supE44 Δ lacU169 (φ80 lacZ ΔM15) hsdR17 recA1 endA1 gyrA96 thi-1 relAI	[27]
*E. coli* DH5a (pUC-*gtf*)	*E. coli* DH5a clone with *gtf*	This study
*E. coli* ET12567	Methylation-deficient *E. coli* dam^-^dcm^-^hsdM	[29]
*Streptomyces sp.* 139	Ebosin producing strain	Lab stock
*Streptomyces* sp. 139 (*ste7*^*-*^)	The gene *ste7* disruption mutant	This study
*Streptomyces* sp. 139 (*gtf*)	Am^s^, thio^r^, the heterologous gene replacement strain of *Streptomyces* sp.139	This study
*Streptococcus thermophilus*	Wild type	CICC 20370
**Plasmids**		
pUC18	Amp^r^, *E.coli* general cloning vector with multiple cloning site	[27]
pUC-*gtf*	Amp^r^, pUC18 derived plasmid carrying *gtf* gene	This study
pKC7	Km^r^, Am^r^, pKC1139 derived plasmid carrying F1, F2	This study
pKC7-*gtf*	pKC7 carrying *gtf* gene	This study
pEGM-T-*tsr*	pEGM-T carrying *thio*^*r*^ gene	Lab stock

### Animal

Kunmin mice (male, 18-20 g, Certificate No. SCXK 2005-0013) and Wistar rats (male, 180 ± 20 g, Certificate No.: SCXK 2005-0013) were purchased from the Institute of Experimental Animals, Chinese Academy of Medical Sciences, Beijing. All rats were housed under standard laboratory conditions with the approval of the Institute of Experimental Animals and Use Committee of Chinese Academy of Medical Sciences.

### Cell culture of FLS (fibroblast-like synoviocytes)

Synovial tissues obtained from the knee joints of sacrificed CIA (collagen- induced arthritis) rats [26] on day 30 after immunization were minced and digested with type II collagenase (0.4%, Gibco) at 37°C in a humidified 5% CO_2_ incubator for 2 h in Dulbecco’s modified Eagle’s medium (DMEM), thoroughly washed and then cultured in DMEM supplemented with fetal bovine serum (15%, Gibco). At confluence, adherent cells were trypsinized (0.25%, Hyclone) at 37°C for 0.5 h, filtered and extensively washed again. The adherent cells were cultured in DMEM containing fetal bovine serum, 100 units/mL penicillin and 100 μg/mL streptomycin in a humidified atmosphere of 5% CO_2_ incubator for 24 h. FLS from passage 3-4 were seeded at 1 × 10^6^/mL in 24-well plates (Nunc) or at 1 × 10^5^/mL in 6-well plates in DMEM and cultivated at 37°C for 24 h.

### DNA isolation and southern blot analysis

Isolation of *E. coli* plasmid DNA, *S. thermophilus* genomic DNA and standard recombinant DNA techniques were performed as described by Sambrook and Russell [27]. *Streptomyces* plasmid and genomic DNA was isolated as mentioned by Kieser et al [28]. For Southern blot analysis, the DIG high prime DNA labeling and detection starter kit II obtained from Roche (USA) was used following the instructions of manufacturer.

### Cloning of the gene *gtf* of *Streptococcus thermophilus*

The genomic DNA isolated from 48-hour cultured *S.thermophilus* was used as template. The *gtf* gene was amplified by PCR using primers P1 and P2 (Table 2) in the following conditions: an initial denaturation at 94°C for 10 min; 30 cycles of 1 min at 94°C, 1 min at 47°C and 3 min at 72°C; and finally 10 min at 72°C. The amplified DNA fragment was cloned into plasmid pUC18 digested with *Eco*RI and *Sal*I to construct pUC-*gtf* . The correct nucleotide sequence of *gtf* gene fragment cloned in pUC-*gtf* was verified by sequencing using an ABI PRISM 377XL DNA Sequencer (Applied Biosystems). Then the recombinant plasmid pUC-*gtf* was transformed into the strain *E. coli* DH5α to produce the strain *E. coli* DH5α (pUC-*gtf*). This plasmid pUC-*gtf* was isolated according the protocol mentioned by Sambrook and Russell [27].

**Table 2:** Oligonucleotide primers used in this study

**Primer**	**Sequence 5′ – 3′**
P1	5′-GCGAATTC TCTAGAATGGCGTGGCTAATTAAATG-3′
P2	5′-GCGTCGAGTTAATCGCTTTCAATA-3′
P3	5′-GCGTCGACAGGCGAATACTTCATATG-3′
P4	5′-GCAAGCTT TCTAGATGATCATCACTGACGAAT-3′

### Construction of the strain *Streptomyces* sp. 139 *(gtf)*

With the plasmid pEGM-T-tsr (unpublished data) as template, the thiostrepton resistant gene (*thio*^*r*^) was amplified by PCR using primers P3 and P4 (Table 2) in the following conditions: an initial denaturation at 94°C for 5 min; 30 cycles of 1 min at 94°C, 0.5 min at 58°C and 2 min at 72°C; and finally 10 min at 72°C. The amplified 1.06 kb gene fragment (*thio*^r^) was inserted into plasmid pUC-*gtf* digested with *Sal*I- *Hin*dIII , which was then digested by *Xba*I to isolate the fragment of thio^r^-*gtf*. During construction of the *ste7* gene disrupted strain *Streptomyces* sp. 139 (*ste7*^***-***^) in previous study [16], the gene *ste7* disruption vector pKC7 containing a 1.03-kb F1 fragment upstream of the gene *ste7* and a F2 0.899-kb fragment downstream of the gene *ste7* was created. The fragment of thio^r^-*gtf* was introduced into the *ste7* disruption vector pKC7 at *Xba*I site to construct the gene replacement plasmid pKC7-*gtf.* Propagated in the methylation-deficient *E. coli* 12567 [29], pKC7-*gtf* was isolated and introduced into *Streptomyces* sp*.* 139 (*ste7*^***-***^) by polyethylene glycol (PEG)-mediated protoplast transformation [28]. Incubated at 28°C for 16 to 20 h, the plates were overlaid with soft R2YE (0.7% agar) containing apramycin (40 μg/mL). Plasmid pKC7-*gtf* bears a temperature-sensitive *Streptomyces* replication origin [20] that is unable to replicate at temperatures above 34°C. Therefore, the transformants were first incubated at 28°C for 2 days until pinpoint size colonies became visible and then shifted to 37°C for further incubation. Strains resulted from a double crossover via homologous recombination grew out of the original pinpoint-size colonies in several days. The heterologous gene replacement strain *Streptomyces* sp. 139 (*gtf*) was selected by both apramycin sensitivity (Am^s^, 40 μg/mL) and thiostrepton resistance (thio^r^, 50 μg/mL).

Five thio^r^ Am^s^ colonies were selected randomly and the isolated genomic DNA originated from *Streptomyces* sp. 139 (*gtf*) and *Streptomyces* sp. 139 (*ste7*^-^) [16] were digested with *Bam*HI individually before DNA blot analysis.

### Isolation of EPSs

The strains of *Streptomyces* sp. 139, *Streptomyces* sp. 139 (*ste7*^-^) and *Streptomyces* sp. 139 (*gtf*) were cultured at 28°C for 96 h respectively. Following the protocol as described before [9], Ebosin, EPS-7 m and EPS-7 g were isolated from the supernatants of fermentation cultures of respective strains.

### Monosaccharide analysis of EPSs

Sugar composition analysis was performed as described by Sun et al [30]. The purified polysaccharide samples (10 mg) was dissolved in 3 ml of 2 M TFA to hydrolyze at 115°C for 3 h, then drying under a stream of nitrogen. Subsequently, the residue was treated with 200 μL of 0.5 M Na_2_CO_3_ at 30°C for 45 min and added 1.5 mL of 4% NaBH_4_ at room temperature for 2 h. After that, the sample was neutralized with 25% acetic acid and loaded onto a cation exchange resin column eluted with H_2_O, then discharged the excess NaBH_4_ with methanol. The sample residue was dried with P_2_O_5_ in vacuum at 85°C for 2 h and acetylated with 2.5 mL of acetic anhydride in 0.5 mL of pyridine at 100°C for 30 min. After cooling, the excess reagent was discharged under a stream of nitrogen and the sample was analyzed by Gas Chromatography (GC, HP5890, HEWLETT, Houston, TX, USA). Galacturonic acid was analyzed using a method based on uronic acid carbazole reaction [31].

### Assay for IL-1R binding activity

The enzyme-linked immune specific assay (ELISA) method reported previously was used to analyze the binding activity for IL-1R of isolated EPSs [15].

### Assay of the enzymatic activity for IL-1β-converting enzyme (ICE)

We found recently that Ebosin is an inhibitor of IL-1β-converting enzyme [32], a key enzyme in synthesis of IL-1β. To assess the inhibitory effects of EPS-7 m [16] and EPS-7 g on ICE enzymatic activity, the assay was conducted according to the protocol reported by koizumi et al [33]. The enzymatic reactions were performed in 100 μL reaction solution consisting of 20 mM HEPES (pH 7.5), 0.1 mM NaCl, 5 mM dithiothreitol, 10% sucrose, 250 μM substrate N-acetyl-Tyr-Val-Ala-Asp- *p*-nitroanilide, 2.0 IU of IL-1β-converting enzyme (ICE), 0.64 ng/μL and 0.128 ng/μL of Ebosin, EPS-7 m and EPS-7 g respectively at 37°C for 45 min.

### Enzyme-linked immunosorbent assays of IL-1β in FLS cells

FLS was seeded at 1 × 10^6^/mL in 24-well plates and cultivated at 37°C for 24 h. Ebosin (3.2-0.128 ng/μL) was added individually to each well and cultivated at 37°C for 1 h, then FLS were stimulated with LPS (5 μg/mL per well) at 37°C for 72 h. IL-1β in the supernatants were analyzed with ELISA kits (Applygen). The cultivated FLS cells un-stimulated with LPS were as contro1, while the cultivated FLS cells stimulated with LPS but not treated by Ebosin were as control 2.

### Treatment of the acute inflamed mice induced by croton oil

The mice were randomly divided into three groups: acute inflammatory mice [34], Ebosin **+** acute inflammatory mice and EPS-7 g **+** acute inflammatory mice (n = 8 in each group). Ebosin and EPS-7 g (100 mg/kg) were orally administered to each mouse in the treatment groups. After 1 h, 50 μL of 2% croton oil (in absolute ethyl alcohol) was smeared on the left auricle of each mouse in three groups for 4 h. All the mice were then sacrificed and ears were excised. The degree of ear edema was measured by weighing deference between the right and left ear.

### Statistical analysis

Data were presented as the mean ± SD from at least 3 independent experiments. The significance of differences between groups was evaluated by Student’s *t*-test. *p* values less than 0.05 were considered significant.

## Abbreviations

EPSs: Exopolysaccharides

FLS: Fibroblast-like synoviocytes

LAB: Lactic acid bacteria

*gtf*: Glucosyltransferase gene

ORFs: Open reading frames

## Competing interests

The authors declare that they have no competing interests.

## Authors’ contributions

ZY carried out bioactivity experiments *in vitro* for EPS-7 g , EPS-7 m, Ebosin and analyzed the primary data. Sugar analysis of EPSs was performed by JS. YB and LB constructed the heterologous gene replacement strain *Streptomyces sp. 139 (gtf)*. RJ, LG and CY isolated EPSs. GC identified anti-inflammatory activity of EPS-7 g and Ebosin *in vivo.* YL designed and conducted the experiments. YL and RZ wrote and revised the manuscript. All the authors read and approved the final manuscript.
